# Evaluation of the effectiveness of a quality improvement intervention to support integration of maternal, child and HIV care in primary health care facilities in South Africa

**DOI:** 10.1186/s12889-020-8397-2

**Published:** 2020-03-12

**Authors:** Lyn Haskins, Jessica Chiliza, Pierre Barker, Catherine Connolly, Sifiso Phakathi, Alison Feeley, Christiane Horwood

**Affiliations:** 1 Centre for Rural Health, University of KwaZulu-Natal, 4th Floor, George Campbell Building, Howard College Campus, Durban, South Africa; 2 Department of Global Health, Boston University School of Public Health, Boston, MA USA; 3 Institute for Healthcare Improvement, 53 State Street, Boston, MA 02019 USA; 4 UNICEF South Africa, Equity House, 659 Pienaar Street, Pretoria, South Africa; 5 MRC/WITS Developmental Pathways Health Research Unit, School of Clinical Medicine, Faculty of Health Sciences, University of the Witwatersrand, Johannesburg, South Africa

**Keywords:** Quality improvement, Integrated care, Maternal health, Child health, HIV/AIDS, South Africa, Growth monitoring, Child growth, Nutrition

## Abstract

**Background:**

Despite policies and guidelines recommending integration of health services in South Africa, provision of maternal and child health services remains fragmented. This study evaluated a rapid, scaleable, quality improvement (QI) intervention to improve integration of maternal and child health and HIV services at a primary health level, in KwaZulu-Natal, South Africa.

**Methods:**

A three-month intervention comprised of six QI mentoring visits, learning sessions with clinic staff to share learnings, and a self-administered checklist aimed to assist health workers monitor and implement an integrated package of health services for mothers and children. The study evaluated 27 clinics in four sub-districts using a stepped-wedge design. Each sub-district received the intervention sequentially in a randomly selected order. Five waves of data collection were conducted in all participating clinics between December 2016–February 2017. A multi-level, mixed effects logistic regression was used to account for random cluster fixed time and group effects using Stata V13.1.

**Results:**

Improvements in some growth monitoring indicators were achieved in intervention clinics compared to control clinics, including measuring the length of the baby (77% vs 63%; *p* = 0.001) and health workers asking mothers about the child’s feeding (74% vs 67%; *p* = 0.003), but the proportion of mothers who received feeding advice remained unchanged (38% vs 35%; *p* = 0.48). Significantly more mothers in the intervention group were asked about their baby’s health (44% vs 36%; *p* = 0.001), and completeness of record keeping improved (40% vs 26%; I = < 0.0001). Discussions with the mother about some maternal health services improved: significantly more mothers in the intervention group were asked about HIV (26.5% vs 19.5%; *p* = 0.009) and family planning (33.5% vs 19.5%; *p* <  0.001), but this did not result in additional services being provided to mothers at the clinic visit.

**Conclusion:**

This robust evaluation shows significant improvements in coverage of some services, but the QI intervention was unable to achieve the substantial changes required to provide a comprehensive package of services to all mothers and children. We suggest the QI process be adapted to complex under-resourced health systems, building on the strengths of this approach, to provide workable health systems strengthening solutions for scalable implementation.

**Trial registration:**

ClinicalTrials.gov NCT04278612. Date of Registration: February 19, 2020. Retrospectively registered.

## Background

Integration of health services commonly refers to the organisation and management of health services in a way that people are able to access comprehensive care when they need it, with a user-friendly, people-centred approach, to achieve efficient and cost-effective coverage of services [1]. At a primary health care (PHC) level, these services should include both preventative and curative services. However, many patients have multiple and complex health needs [2] and integrating health services must balance the advantages of integration, with the resulting demands this may place on service-delivery systems.

In South Africa (SA), despite several policies supporting integration of PHC services such as the Ideal Clinic Realisation and Maintenance programme (ICRM) [3] and PHC Re-engineering [4], services for mothers and children are often fragmented, and essential services for mothers and children are frequently provided by different health practitioners in different areas of the clinic [5]. Furthermore, the physical structure of the clinic can be a limitation. Fragmentation of services leads to missed opportunities to provide comprehensive care to mothers and children, increased cost for the patient in transport and time off work, increased cost for the health system, and poor health outcomes [5]. To improve health delivery, it is important that health systems use existing resources more effectively and efficiently to improve coverage of health services and retention in care, and to improve outcomes for mothers and children [6].

Quality Improvement (QI) is a simple, low-tech, systematic, data-driven approach that may lead to measurable improvement in health care services and the health status of targeted patient groups [7, 8]. The QI methodology used was adapted from the Institute for Healthcare Improvement (IHI) breakthrough series (BTS) model [9], which has been successfully used in several South African healthcare settings [10, 11]. Several QI approaches have been used. During QI implementation, routinely collected data is analysed to identify service gaps, and solutions are developed and tested to address these issues. Limitations to QI that have been identified include that this approach may be resource intensive, require ongoing effort for at least 12–18 months and long-term funding, and may take time away from other job responsibilities [12].

In South Africa, QI has been successfully used to improve the quality of prevention of mother-to-child transmission (PMTCT) programmes in health facilities in KwaZulu-Natal (KZN) province [13], becoming a template for PMTCT national scale up in South Africa [14]. In addition, QI has been used in community settings [15] and has been shown to significantly increase exclusive breastfeeding among mothers served by community health workers (CHWs) exposed to a QI intervention [16]. Results from QI interventions using non-randomised designs have been criticized as being methodologically weak [13].

A stepped wedge trial provides a robust methodology for evaluation when a phased approach to implementation is appropriate [17, 18]. This paper describes a stepped wedge evaluation of a QI intervention designed to improve the integration of maternal and child health (MCH) and HIV services with well mother-baby services within public health services in one district in KZN, using a time-limited intervention (three -month) in a complex environment.

## Methods

### Study setting

The study was undertaken in one district in KZN comprising five sub-districts, with a population of approximately 669,000 and with over 147,000 households in the district. The sub-districts vary in size from the largest with a population of 237,500, to the smallest with 83,000 people [19]. Unemployment is high in this district, ranging from 34 to 57% in different sub-districts. More than half of the population in the district lives on less than US$20 per month [19]. The average proportion of households with access to potable water is 77% and access to electricity is 70% [19].

Health care services are provided by 37 primary health clinics, one community health centre, two district hospitals and one regional hospital [20]. The district has a high tuberculosis incidence of 533/100,000 in 2015/16 [15] and an antenatal HIV prevalence of 36.3% in 2015 [21]. Immunisation coverage for children under 1 year of age was 85% for 2015/16, and there was a high in-patient case fatality rates from severe acute malnutrition among children aged under 5 years [20]. At the time of the study, nutrition and growth monitoring for children aged under 5-years was a key priority of the SA National Department of Health.

### Description of the intervention

The QI intervention was structured as a BTS collaborative, a peer-to-peer learning model designed to improve system performance through structured data-driven improvement activities tied to a knowledge-sharing network [9]. This was implemented in 27 clinics in four sub-districts in one district in KZN over a one-year period. In PHC clinics, care is provided by a team of health workers including an operational (clinic) manager, specialist PHC nurses, registered nurses, enrolled nurses, nutritional advisors and lay counsellors. The number of nurses deployed in a clinic is determined by the size of the catchment population.

**Fig. 1:**
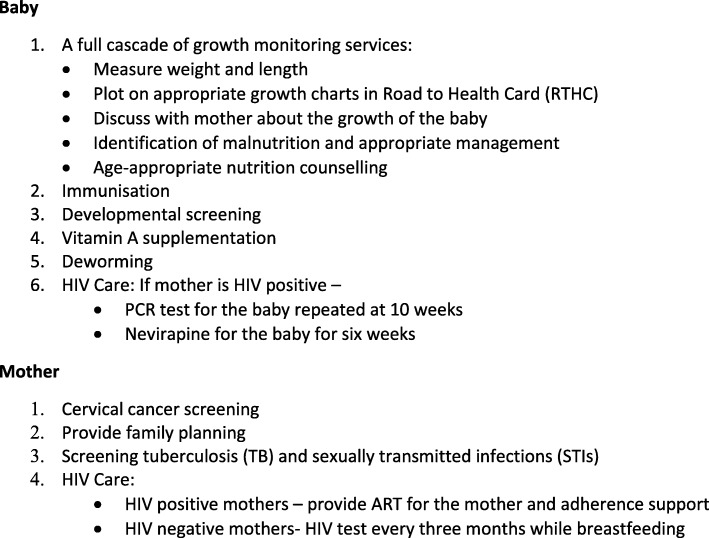
Comprehensive Package of Maternal, Child and HIV Health care. A description of integrated health care services health workers were expected to provide to mothers and babies at each visit to the clinic

There were between five and 10 clinics per sub-district (Table 1) and the intervention was conducted at the same time in all clinics in each sub-district. All participating clinics provided the same package of health services in compliance with Department of Health policies, which includes all services for mothers and children set out in the integrated package of care (Fig. 1). The fifth sub-district was used to pilot the methodology and was not included in the evaluation. The QI intervention aimed to support provision of an integrated service for mothers and children attending immunization services. We will refer to this integrated service as a ‘well mother-baby service’.

**Table 1:** Interviews conducted during each wave

		Wave 1 (Baseline)	Wave 2	Wave 3	Wave 4	Wave 5 (Endpoint)
**Data Collection**		**Dec 2015-Jan 2016**	**Mar-Apr 2016**	**June–July 2016**	**Sept-Oct 2016**	**Dec 2016-Feb 2017**
**Intervention Period**			**Jan- Mar 2016**	**Apr-June 2016**	**July-Sept 2016**	**Oct 2016-Jan 2017**
	**Clinics**	**Interviews**	**Interviews**	**Interviews**	**Interviews**	**Interviews**
**Sub-district 1**	10	108	106^*^	103^*^	107^*^	104^*^
**Sub-district 2**	6	63	66	58^*^	62^*^	63^*^
**Sub-district 3**	6	74	64	67	62^*^	62^*^
**Sub-district 4**	5	60	52	53	55	55^*^
**Total**	27	305	288	281	286	284

* Number of interveiws post QI intervention

The aim of the intervention was to ensure that all mothers and children attending the clinic received a comprehensive package of services at every visit. Integration was defined as mother-baby pairs receiving all required health services contained in a defined package of services at every visit. Services included in the integrated package are shown in Fig. 1. Growth monitoring comprises a series of activities conducted sequentially, all of which need to be completed for growth monitoring to be effective (Fig. 1).

Implementation of the intervention in participating clinics was facilitated by a quality mentor (QM) employed by the project, who was a PHC nurse trained in QI techniques and methodology. The intervention comprised of a series of five QI mentoring visits per clinic conducted every 2 weeks in a single sub-district over a three-month period. Before the intervention commenced in the sub-district, staff who provided maternal and child health services from all clinics attended a learning session to introduce the project, and another learning session was conducted on completion of the five mentoring visits. After completion of the three-month intervention, a final mentoring visit (sixth visit) was conducted with the local clinic supervisor to hand over QI activities, which served as a close out for the intervention. Data on coverage of the key integrated package of services was monitored over the three-month period of intervention in each clinic using a self- administered checklist. A self-administered checklist was used in each clinic over the three-month period of implementation, this served a dual purpose of prompting health workers to implement the package of services as well as monitoring progress of implementation.

#### Clinic mentoring visits

The first mentoring visit was conducted as a short workshop attended by all members of the clinic team who provided services to mothers and children and served as an introduction to the project. During the workshop, participants drew a map of the clinic to show how, where and by whom, MCH and HIV services were provided. Clinic staff were asked to critically assess current service provision for mothers and children and identify service gaps to be addressed to improve integration of MCH services (Fig. 1). A clinic QI team was convened from among clinic staff providing MCH services, to support the provision of integrated services during the project intervention period and beyond. The clinic QI team included the operational manager, an all health workers in the well mother-baby clinic, community health workers and data support staff.

Four subsequent mentoring visits were conducted over the three-month intervention period, each lasting 2–3 h. The QM undertook a series of activities to monitor integration of services, including: observing consultations with mothers and babies; conducting exit interviews (Supplementary File 1) with mothers; and reviewing children’s patient-held records (Road to Health Booklet; RTHB) to assess the number of services received on the day of the visit. Mentoring tools were used to support these activities, including a RTHC review checklist and an exit interview guide (Supplementary File 1). The QM met with the clinic QI team to provide feedback on these activities, review improvement plans and monitor progress towards providing an integrated well mother-baby service. QI activities successfully undertaken to improve integration included: streamlining patient flow for mothers and children; moving growth monitoring into the consulting room; providing all MCH services together in one consulting room; ensuring that all mother-baby pairs were seen by registered nurses, and ensuring that all equipment and supplies were readily available. In most clinics this meant that the comprehensive services were provided in a single MCH consulting room where health workers with different scopes of practice worked together.

The QM also provided in-service training to staff to address knowledge gaps identified by staff. A sixth visit was conducted after completion of the mentoring process, during which all tools and activities were handed over to the clinic supervisor in the district to enable continuation of the project activities.

#### Learning sessions

Learning sessions were conducted each quarter as implementation was completed in one sub-district and started in the next sub-district to share and review the successes and challenges, encourage peer-to-peer learning and support health facility staff. Each learning session was attended by health workers from all clinics in two sub-districts, that is the sub- district where the intervention was wrapping up and the sub-district where it was just starting. Thus, clinic staff from each sub-district attended two learning sessions. Five learning sessions were conducted in total, one at the start of project and from then on every 3 months over the 1 year implementation period.

**Fig. 2:**
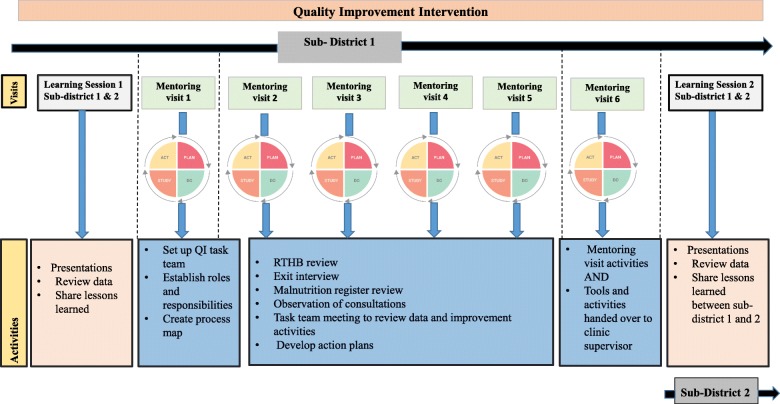
Description of Study Intervention. Outline of quality improvement intervention activities roll out in each sub-district

Relevant members of the district management team attended the learning sessions to promote participation, buy-in and support for the project at district management level (Fig. 2).

#### Self-administered checklists

Self-administered checklists were used to generate the data to drive the quality improvement process. The data required to monitor ongoing progress towards providing integrated services was not routinely available. Although relevant routine indicators (e.g. HIV testing, family planning etc.) were collected in clinics, these did not distinguish between services provided during an integrated well mother-baby visit and those provided at separate visits. Therefore, a self-administered checklist was developed which included all the elements of an integrated mother-baby service (Fig. 1). Health workers completed these checklists during the consultation to track the services mothers and babies received during well mother-baby clinic visit and identify the gaps in providing comprehensive services. Checklists also served as a reminder about services to be offered in the integrated well mother-baby package. Completed checklists were collected by the project team and data was collated and fed back to the clinic QI team at subsequent mentorship visits. Progress during the intervention period was reviewed at the learning sessions.

### Study design

A stepped wedge study was conducted to quantitatively evaluate the QI intervention using a phased approach. A stepped wedge design is a type of cluster randomised trial, where clusters are randomised and systematically and sequentially exposed to the intervention and evaluated over time [17]. Ethically appropriate, well-designed, stepped wedge studies can provide evidence of the effects of interventions and are considered higher quality than evidence from non-randomised studies [17, 18].

The stepped wedge design starts with a baseline assessment with no clusters exposed to the intervention, followed by each cluster sequentially randomised to cross from control group to intervention group at regular intervals, until all clusters are implementing the intervention [17]. In contrast to randomised controlled trials, this design allows the benefits of providing the intervention to include all clusters [17]. The stepped wedge design allows every cluster (in this case all clinics in a single sub-district) to provide pre and post intervention data over an extended period of time [17]. This is a strong research design for implementation research where it is not feasible or acceptable to have a control group that does not receive the intervention.

### Sample size

The unit of randomisation was the sub-district. The order in which sub-districts received the intervention was randomly allocated from a list of four sub-districts. The intervention was implemented consecutively in this order in each sub-district over a three-month period. All clinics in one sub-district participated in the study and received the intervention at the same time (Table 1).

The sample size was calculated to achieve 80% power to detect a 13% change in the proportion of mother-baby pairs who received a full package of care, from a non-informative baseline level of 50%. In order to take account of the clustering effect of mother’s attending the same clinic, a random effects multi-level mixed logistic model was used where the clinic was the random effect. The ICC (intra-class correlation) of 0.05 was included in the sample size calculation to adjust for clustering.

Ten mother-baby pairs were enrolled in each of the 27 participating clinics, at each wave. Therefore, the total sample size was 270 mother-baby pairs for each wave of data collection, giving a total of 1350 participating mother-baby pairs after completion of five waves.

### Data collection

Data are collected in five waves (Table 1) every three-months over the one-year intervention period. Data for the first wave was collected before starting implementation, where all clinics functioned as controls, and served as a baseline. Further waves of data collection continued as each sub-district completed the intervention, until the last wave of data collection when the intervention was complete in all sub-districts.

Sequential sampling was used to recruit mother-baby pairs and all mothers attending the clinic for a well mother-baby visit with a child under the age of 24 months were approached to participate. This was continued until the sample size was achieved. An exit interview with participating mothers was conducted by trained fieldworkers, using a structured data collection tool developed specifically for this study on electronic tablets. The exit interview included questions regarding the maternal, child and women’s health and HIV services received during the visit. Children’s patient-held records (RTHBs) were reviewed to identify which services had been received that day and whether all health services for babies were up to date. Data collection continued in each clinic until the desired sample was reached.

### Data analysis

A multi-level mixed logistic regression model adjusting for clustering was used for analysis. to take account of random cluster (clinic) effects and a fixed time and group effect [22]. The unit of analysis was the mother-baby pair. Data was analysed using STATA V13.1. Each element of comprehensive package of care was designated as provided if the mother reported having received it (exit interview), or if it was recorded as being given on the patient held record (RTHB review). These data are presented separately, we did not combine findings from the exit interview and RTHB review.

Overall differences in demographic characteristics between the control and intervention groups at baseline were compared using chi square or Mann –Whitney tests. The overall proportion of women with a positive result in each group (intervention and control) over the full period is reported, as well as the odds ratio and *p* value from the model. *P* value < 0.05 was considered to be statistically significant.

## Results

The study was conducted between January 2016 and February 2017. In total, 1441 exit interviews were conducted over five waves of data collection in 27 participating clinics (Table 1). Socio-demographic details of study participants showed some difference between baseline and control (Table 2).

**Table 2:** Sociodemographic characteristics of study participants

	Intervention ***n*** = 777		Control ***n*** = 661		Total ***n*** = 1438		***p***-value
**DEMOGRAPHICS**	**median**	**IQR**	**median**	**IQR**	**median**	**IQR**	
**Baby’s age (months)**	5.0	(2.0–9.0)	5.0	(2.0–9.0)	5.0	(2.0–9.0)	0.6
**Mother’s age (years)**	25.0	(21–31)	25.0	(21–30)	25.0	(21–31)	0.7
**BABY DEMOGRAPHICS**							
**Baby’s age group**							
< 6 months	408	52.5%	344	52.0%	752	52.3%	**0.04**
6–11.9 months	252	32.4%	210	31.8%	462	32.1%	
12–17.9 months	97	12.5%	71	10.7%	168	11.7%	
> =18 months	20	2.6%	36	5.4%	56	3.9%	
Total	777	100.0%	661	100.0%	1438	100%	
**Sex**							
Male	378	48.6%	334	50.5%	712	49.5%	0.5
Female	399	51.4%	327	49.5%	726	50.5%	
Total	777	100.0%	661	100.0%	1438	100%	
**Living in same HH**^1^							
Yes	744	95.8%	644	97.4%	1388	96.5%	0.084
No	33	4.2%	17	2.6%	50	3.5%	
Total	777	100.0%	661	100.0%	1438	100%	
**MOTHER’S DEMOGRAPHICS**							
**Mother’ age**							
< 20	99	12.8%	91	13.8%	190	13.2%	0.7
20–24	260	33.5%	203	30.8%	463	32.2%	
25–29	192	24.7%	169	25.6%	361	25.2%	
30–46	225	29.0%	197	29.8%	422	29.4%	
Total	776^a^	100.0%	660	100.0%	1436	100.0%	
**Mother’s education**							
grades 2–7	34	4.4%	24	3.7%	58	4.0%	**0.03**
grades 8–11	297	38.2%	295	45.0%	592	41.3%	
grades 12+	446	57.4%	337	51.4%	783	54.6%	
Total	777	100.0%	656	100.0%	1433	100.0%	
**Mother currently working**							
Yes	139	18%	93	14.1%	232	16.1%	**0.05**
No	638	82%	568	85.9%	1206	83.9%	
Total	777	100%	661	100.0%	1438	100.0%	

^a^missing DOB

Coverage of many elements of growth monitoring improved significantly from between control and intervention groups (Table 3), this included the length of the baby being measured (77% vs 63%; *p* = 0.001), and recorded in the RTHB (66% vs 43%; *p* = 0.001). Although the proportion of children with a fully completed RTHB improved, this remained low in intervention clinics (40% vs 26%; *p* = 0.001). Health workers were more likely to have asked mothers about the child’s feeding in intervention clinics (74% vs 67%; *p* = 0.003), but the proportion of mothers who received feeding advice was low and no improvement was shown (38% vs 35%; *p* = 0.48).

**Table 3:** Growth monitoring activities completed

	Intervention		Control		OR	***P***-value
	N	n (%)	N	n (%)		
**From Exit Interview**						
Was your baby weighed today?	777	773 (99%)	661	651 (99%)	over 95% at baseline unable to model	
Was your baby’s length measured?	777	599 (77%)	661	419 (63%)	**2.4 (1.4–3.9)**	**0.001**
Shown growth on growth chart	777	378 (49%)	661	295 (45%)	1.5 (1.0–2.2)	0.05
Mother reports being asked how she is feeding the baby	777	578 (74%)	661	444 (67%)	**1.9 (1.3–3.0)**	**0.003**
Mother reports being given advice about feeding the baby	777	296 (38%)	661	233 (35%)	1.2 (0.8–1.8)	0.48
**From RTHB Review**						
Weight recorded today	776^a^	735 (95%)	658^a^	585 (89%)	1.7 (0.8–3.6)	0.2
Length recorded today	776^a^	514 (66%)	658^a^	282 (43%)	**2.2 (1.4–3.6)**	**0.001**
Weight for age plotted today	776^a^	713 (92%)	658^a^	610 (93%)	1.1 (0.5–2.2)	0.8
Weight for length/height plotted today	776^a^	392 (51%)	658^a^	289 (44%)	**2.0 (1.3–3.0)**	**0.001**
Diagnosis for nutrition recorded in RTHB	776^a^	518 (67%)	658^a^	229 (35%)	**2.3 (1.5–3.5)**	**< 0.0001**
All nutrition elements completed in RTHB	776^a^	312 (40%)	658^a^	174 (26%)	**2.1 (1.3–3.4)**	**0.001**

^a^missing data because no RTHB available

Coverage of several other individual services in the comprehensive package of care for children was high at baseline and did not show improvement. Significantly more health workers in the intervention clinics provided a complete package of care for children (comprising growth monitoring, advice about feeding, developmental screening, vitamin A supplementation, deworming and immunization if they were due) compared to control clinics (14.7% vs 6.5%; p = 0.003) although the proportion of children receiving all services remained low.

For maternal health services, coverage remained poor. Although health workers were more likely to ask about family planning (34% vs. 20%; *p* <  0.001) and HIV testing (27% vs 20%; *p* = 0.009), this did not result in an increase in the number of mothers receiving these services on the day of the clinic visit (Table 4).

**Table 4:** Maternal and child health activities undertaken and services received at this visit

	Intervention		Control		OR	P
	N	n (%)	N	n (%)		
**Child health activities and services**						
**Developmental Screening**						
Mother reported being asked about the baby’s health	777	340 (43.8%)	661	235 (35.6%)	2.0 (1.4–3.0)	**0.001**
Mother reported being asked about how the baby is developing	777	209 (26.9%)	661	200 (30.3%)	1.2 (0.8–1.8)	0.43
Developmental screening is up to date in RTHB^a^	534^a^	354 (66.3%)	458^a^	279 (60.9%)	1.8 (1.1–3.0)	**0.017**
**Vitamin A**						
Vitamin A is up to date for age in RTHB^b^	371^b^	335 (90.3%)	320^b^	290 (90.6%)	0.9 (0.3–2.8)	0.95
**Deworming**						
Deworming is up to date for age in RTHB^c^	117^c^	85 (72.6%)	107	73^c^ (68.2%)	1.5 (0.5–4.8)	0.45
**Immunisation**						
Immunisation is up to date for age in RTHB^d^	**754**	744^d^ (98.7%)	640	623^d^ (97.3%)	2.1 (0.9–4.9)	0.09
**Maternal health activities and services**						
**HIV Care**						
Mother reports being asked about HIV today	777	206 (26.5%)	661	129 (19.5%)	1.8 (1.2–2.9)	**0.009**
*Number of HIV negative/HIV unknown mothers*	*N* = 463		*N* = 381			
HIV negative mothers report being up to date for HIV retesting today (repeated 3 monthly)	463	264 (57.0%)	381	183 (48.0%)	1.1 (0.7–1.7)	0.60
*Number of HIV positive mothers*	*N* = 314		*N* = 280			
HIV positive report being on ART	314	312 (99.4%)	280	270 (96.4%)	2.7 (0.3–24.9)	0.87
HIV positive mothers ART indicated today^e^	312	171 (54.8%)	270	144 (53.3%)	1.0 (0.6–1.8)	0.9
HIV ART given today	171	116 (67.8%)	144	84 (58.3%)	1.1 (0.7–1.7)	0.6
**Family Planning**						
Mother reports being asked about family planning	777	260 (33.5%)	661	129 (19.5%)	5.3 (3.2–8.8)	**< 0.001**
Mother reports being asked if she needed condoms	777	105 (13.5%)	661	60 (9.1%)	3.0 (1.5–6.3)	**0.003**
Condoms given today	777	131 (16.9%)	661	60 (9.1%)	3.3 (1.7–6.5)	**< 0.001**
*On oral or injectable family planning method*	*N* = 595		*N* = 499			
Family planning (oral/injection) visit due within 14 days = FP due today	595	188 (31.6%)	595	117 (23.5%)	1.6 (1.0–2.3)	0.03
FP given today	188	76 (40.4%)	117	49 (41.9)	1.1 (0.6–2.1)	0.8
**Cervical Cancer Screening**						
Mother reports being asked about having a PAP smear	777	67 (8.6%)	661	60 (9.1%)	1.4 (0.5–3.9)	0.46
HIV positive mothers where cervical screening is indicated (i.e.no PAP smear in last 12 months)	*N* = 246		*N* = 249			
PAP smear done today on HIV+ mother where indicated	246	3 (1.2%)	249	1 (0.4%)	Unable to calculate	
**STI Screening**						
Mother reports being asked whether she has vaginal discharge or sores	777	84 (10.8%)	661	54 (8.2%)	2.0 (0.8–5.0)	0.12
**TB Screening**						
Mother reports being asked whether she is coughing	777	117 (15.1%)	661	80 (12.1%)	1.6 (0.8–3.0)	0.19

^a^Developmental screening starts at 14 weeks therefore children < 14 weeks of age were excluded resulting in the denominators indicated

^b^Vitamin A supplementation starts at 6 months therefore children < 6 months of age were excluded resulting in the denominators indicated

^c^Deworming starts at 12 months therefore children < 12 months of age were excluded resulting in the denominators indicated

^d^Immunisation starts at 6 weeks therefore children < 6 weeks of age were excluded resulting in the denominators indicated

^e^ART is indicated today if next appointment is within the next 7 days

## Discussion

To our knowledge this is the first rigorous evaluation of a QI intervention where multiple indicators were used to document wide-ranging changes to the provision of maternal and child health services in a PHC setting. Although we were able to achieve significant improvements of coverage in some services, we were unable to make substantial changes required to provide a comprehensive package of services to all mothers and children. Integration of health services is increasingly acknowledged as pivotal to improving the coverage of interventions, the efficiency and effectiveness of service provision, and this is supported by several key national health policies in SA, including the ICRM Programme [3] and PHC re-engineering [4]. However, thus far, no feasible approach has been evaluated to operationalise integrated services, and implementation remains poor.

We showed significant improvements to many growth monitoring elements, but we were not successful in improving coverage of additional MCH services. There was an increase in the proportion of consultations where several components of the integrated service were discussed, suggesting awareness of these services improved among health workers and mothers but no increase in coverage of services. For example, mothers attending intervention clinics were more likely to have discussed family planning and HIV testing during their visit, although this did not result in an increase in provision of those services. A likely explanation is that it was easier for health workers to improve coverage of existing well-established services, like growth monitoring, than to achieve the widespread re-organisation of equipment and human resources required for the addition of previously unavailable services their settings.

One major challenge to providing an integrated service is the current inflexible dispensing practices. Health workers are unable to provide additional medication or dispense medication before the date it is due. It is currently the norm for mothers to receive different services on different days of the month (e.g. family planning, antiretroviral therapy (ART) and immunisations), and there is no mechanism available for health workers to align future appointments to enable a mother to attend the clinic and receive all medications on a single visit. This is a major administrative barrier to achieving integrated service provision and is the result of strongly established practices in PHC service provision, which will require policy changes within the Department of Health if it is to be resolved. Until provision of integrated services becomes a defined target, and workable solutions are implemented to align clinic appointments, integration will remain very difficult to achieve.

QI is accepted as a simple and effective strategy for health systems strengthening [23] and has been widely and successfully implemented in several health settings in low-resource countries [24, 25]. QI is potentially a powerful approach to scale up innovations and create learning networks, but robust evidence of long-term effectiveness remains sparse and interventions are often resource intensive [12]. Most QI initiatives have used a small number of routinely collected data elements, and rapid cycle tests of change (plan-do-study-act), with available data used to monitor the success of multiple small improvement actions, and to monitor actions shown to be successful when they are rapidly rolled out. However, if QI is to provide the key to health system strengthening, it needs to be flexible and adaptable to address a wide range of health system challenges, rather than confined to those problems where easily accessible, simple indicators are available to drive change. The over-arching principle that QI interventions should be data-driven is a key strength of the approach but is also a major challenge that needs further exploration and evaluation. Improving the quality of data is one of the first steps when starting a QI project, which sometimes makes it difficult to distinguish between improvements to data quality and genuine improvements to health services. If successful improvement actions are to be identified and rapidly rolled out, data on key indicators needs to be available quickly to QI teams, but this may not be achievable in low resource settings or where multifaceted system problems are being addressed, thus limiting the applicability of the QI approach. While there is growing evidence that QI contributes to health system change, broader change is often needed, including both the need for system change as well as provider behavior change, which are complex challenges.

This project used a composite indicator to drive implementation in the clinic, this was the proportion of mothers and babies receiving a full package of care, and this indicator was generated from a number of data elements that are not routinely available. We addressed this challenge by collecting data using a self-administered checklist, which HWs completed during each mother-baby consultation. This aimed to serve as a reminder to HWs, as well as a source of data to monitor success of QI activities. Observation checklists have been used successfully in other settings to support QI interventions, when administered by trained QI facilitators [26]. However, in our study, this approach was largely unsuccessful. Despite ongoing encouragement and support, HWs were often failed to complete the checklist, or did not complete it accurately. Further HWs lacked the skills to collate the checklist data themselves, as a result the checklists did not inform or guide the intervention as intended. The primary reason given for this was that completing the checklists was time consuming. Providing training ahead of implementation followed by supervisory support has been shown to improve uptake of quality improvement checklists in other settings [27]. Using project staff collect and collate the data was challenging and costly in the rural setting where we worked and resulted in clinic staff no longer having ownership of the data. Therefore, we suggest that innovative solutions such as setting up practical systems to collect and manage data, and training for HWs to interrogate their data and provide feedback, are necessary if QI is applied to health system challenges where data for guiding improvement actions is not easily available.

Many QI initiatives depend on specialist QI practitioners or skilled facilitators who are involved with the intervention for prolonged and often open-ended time periods [12]. Our objective was to determine whether QI principles could be implemented in a complex environment, using a highly structured intervention designed to be used by existing PHC supervisors over a relatively brief time period. Although our facilitator had received a short training in QI, he was not a QI specialist. If QI is to be widely applicable and implemented at scale in low resource settings, it is essential that it can be successfully implemented with existing facilitators/supervisors or managers with short additional training. We developed a range of tools to support the mentoring process and make the QI process more structured and less dependent on individuals’ skills, and therefore replicable in different settings. Further evaluation is required to determine the success of QI interventions when integrated into routine management activities at scale.

Another key pillar of QI is the creation of strong peer support systems who own the intervention and learn together during regular learning sessions [12]. Peer learning can be a game changer in improving HW practice but was challenging in our setting because of logistical difficulties to bringing clinic teams together from geographically dispersed rural clinics. Additionally, service provision is disrupted when teams are removed from the clinic settings. As a result, only five learning sessions were conducted over the one-year implementation period, and each group of clinic staff only attended two learning sessions at the start and end of the implementation period in their sub-district. QI processes may need further adaptation to ensure strong learning networks are established in low-resource settings. It may be possible to use technology to convene teams or use a smaller learning network (comprising two or three clinics) to replace large-scale learning sessions. Finally, existing district meetings could function as learning sessions which would provide an ideal way forward for these meetings to become a routine platform for district administrators and supervisors to ensure ongoing peer learning and support.

### Strengths and limitations

This study employed a robust methodology, where all participating facilities contributed to both intervention and control groups. Limitations to the methodology include that data relied on reports from mothers and from recording interventions from the clinical records and were not observed directly. In addition, there was no assessment of quality and appropriateness of interventions provided. Further, the additional paperwork required by the intervention may have had a negative impact on quality of care, thus reducing the effect of the intervention.

Stepped wedge studies are less affected by variabilities of each cluster (or clinic) since each cluster contributes to the control and intervention data during the study [28], and we were able to learn from each intervention period and modify the intervention based on the previous sub-district’s experience [29]. While stepped wedge designs are known to be a rigorous research design and can produce strong evidence, they do pose various challenges. Logistically, such studies can be time intensive with repeated trainings and more administrative work required as intervention groups join the study [30].

## Conclusion

This rigorous evaluation demonstrated that implementation of a time-limited QI strategy effectively improved coverage of some components of an integrated maternal and child health service in a complex health environment, but we were unable to achieve the changes needed to provide a comprehensive package of care for mothers and children. However, we suggest that key QI processes should be adapted to address the challenges of implementing QI in complex, under-resourced health systems using available human resources. In addition, higher level health system strengthening interventions may be required to support QI processes at clinic level. Further research is required to evaluate the performance of QI in such settings at large scale.

## Supplementary information

**Additional file 1.** Exit Interview Guide. A standardized set of question used to guide the interview with study participants.

## Data Availability

The datasets used and/or analysed during the current study are available from the corresponding author on reasonable request.

## Abbreviations

ART: Antiretroviral Therapy
BTS: Breakthrough series
CHW: Community Health Worker
HIV: Human Immunodeficiency Virus
HWs: Health Workers
ICC: Inter Cluster Correlation
ICRM: Ideal Clinic and Realisation and Maintenance
IHI: Institute for Healthcare Improvement
KZN: Kwa-Zulu Natal
LMIC: Low and Middle Income Countries
MCH: Maternal and Child health
PCR: Polymerase Chain Reaction
PHC: Primary Health Care
PMTCT: Prevention of Mother-to-Child transmission
QI: Quality Improvement
QM: Quality Manager
RTHB: Road to Health Booklet
SA: South Africa
