# Supplementary material for: Evaluation of the effectiveness of a quality improvement intervention to support integration of maternal, child and HIV care in primary health care facilities in South Africa
Source: BMC Public Health. 2020 Mar 12;20:318. doi: 10.1186/s12889-020-8397-2 (PMC7069172; doi:10.1186/s12889-020-8397-2)
Supplement: Supplementary file 1 — Additional file 1. Exit Interview Guide. A standardized set of question used to guide the interview with study participants. [file 12889_2020_8397_MOESM1_ESM.pdf]

C☐☐M☐☐☐

INTEGRATED NUTRITION PROJECT – EXIT INTERVIEW WITH MOTHERS

**AD1.** Date of interview	D D M M Y Y Y Y	**AD2.** Interviewer ID	
**AD3.** Sub-District code		**AD4.** Clinic name /code	*Pre-filled*
**AD5.** Wave	*Pre-filled*	**AD6.** Time of arrival at the clinic (from screening form)	☐☐:☐☐ AM / PM
**AD7.** Time of interview starting	☐☐:☐☐ AM / PM		

C☐☐ M☐☐☐

SECTION A – DEMOGRAPHIC (DEM)				
**Interviewer: The following questions are about your child, yourself and your family**				
A1	What is your date of birth?<br>**Usuku lwakho lokuzalwa (mother)?**	☐☐/☐☐☐/☐☐☐☐		
A2	Childs date of birth<br>**Usuku lomntwana lokuzalwa**	☐☐/☐☐☐/☐☐☐☐		
A3	What is the age of the child?<br>**Unezinyanga ezingaki umntwana?**	☐☐ weeks OR	☐☐ months	
A4	What is the sex of this child?<br>**Buthini ubulili bomntwana wakho?**	1 = Male	2 = Female	
A5	Are you living in the same household as this child?<br>**Uhlala naye lomntwana?**<br>*Note should be sleeping in the same house at least 4 nights per week*	1 = Yes	2 = No	
**Interviewer: I am now going to ask you some questions about the father of this child:**				
A6	Is the father of this child alive?<br>**Ngabe usaphila ubaba wontwana?**	1= Yes<br>2= No → **skip to A11**<br>3 = Do not know → **skip to A11**		
A7	Does the father of this child live in the household with you?<br>**Uhlala layikhaya ubaba womntwana?**	1 = Yes	2 = No	
A8	Is the father of this child working?<br>**Ngabe uyasebenza ubaba womntwana?**<br>*Note if the father does any work respond yes*	1 = Yes	2 = No → skip to A10	3= DNK → skip to A10
A9	Is the father working full time or part-time?<br>**Ngabe ubaba womntwana usebenza full time noma part time?**<br>*Note full time is for the whole day at least 5days per week, anything less is part-time*	1 = fulltime	2 = part-time	
A10	In the past three months has the father contributed to support the child?<br>**Ezinyangeni ezintathu ezindlule kukhona okunikezwe ubaba womntwana ukondla umntwana?**<br>*Note could be giving cash or buying things for the child. Regularly means every week or month at regular intervals*	1 = regularly<br>2 = sometimes<br>3 = not in the past 3 months		
**Interviewer: I am now going to ask some questions about yourself**				
A11	What is the highest level of school you completed?<br>**Iliphi ibanga oliphumelele esikoleni?**<br>*Note post school qualification should be a formal degree or diploma (at least a year studying) and should be* ***completed.***	0 = None<br>1 = Grade<br>2 = Grade 2<br>3 = Grade 3<br>4 = Grade 4<br>5 = Grade 5<br>6 = Grade 6	7 = Grade 7<br>8 = Grade 8<br>9 = Grade 9<br>10 = Grade 10<br>11 = Grade 11<br>12 = Grade 12<br>13 =Post school qualification	
A12	Are you currently employed or working?<br>**Ngabe uyasebenza?**<br>*Note if she does any paid work- respond YES.. If she is currently on maternity leave and intends to go back to her job respond YES*	1 ○ Yes	2= No<br>→ **Skip to A16**	

C☐☐ M☐☐☐

A13	Are you employed full time or part-time?<br>**Ngabe usebenza full time noma part time?**<br>*(Note full time is for the whole day on at least 5days per week, anything less is part-time.)*	1 = fulltime	2 = part-time
A14	Do you receive a printed payslip every month?<br>**Ngabe uyayithola i payslip njalo ngenyanga?**	1 = yes	2 = no
A15	Did you have to take a day off work to bring your child to the clinic?<br>**Ngabe bekudingeke ukuthi ungayi emsebenzini ukuze ulethe umntwana wakho ekliniki?**	1 ○ Yes	2○ No
A16	How did you get to the clinic today?<br>**Ufike kanjani ekliniki namhlanje?**	**1** ○ Walked → **Skip to B1**<br>2 ○ public transport (bus or taxi)<br>3 ○ private transport eg car<br>4 ○ Other - specify	
A17	How much did it cost you to come to the clinic today? (one way)<br>**Kubize malini ukuza lana eclinic namuhla?**<br>*Note write 000:00 if there was no cost. If the cost is unclear eg petrol in own car write 999:99*	☐☐☐ : ☐☐ Rands	

C□□M□□□

SECTION B. SERVICES PROVIDED			
**INTERVIEWER: I AM GOING TO ASK YOU ABOUT WHETHER YOU AND YOUR BABY WERE OFFERED SPECIFIC SERVICES AT THE IMMUNIZATION CLINIC TODAY**			
B1	Today was your baby weighed?<br>**Ukaliwe namhlanje umntwana wakho?**	1 = Yes	2 = No
B2	Today was your baby's length measured?<br>**Babukalile namhlanje ubude bomntwana wakho?**	1 = Yes	2 = No
B3	Today were you shown on the growth chart how your baby is growing?<br>**Bakutshengisile namhlanje ekhadini elitshengisa ukukhula komntwana ukuthi umntwana wakho ukhula kanjani?**	1 = Yes	2 = No
B4	Today were you asked how you are feeding the baby?<br>**Bakubuzile namhlanje ukuthi umntwana wakho umdlisa kanjani noma umncelisa kanjani?**	1 = Yes	2 = No
B5	How are you feeding your baby now?<br>**Udla ini umntwana wakho manje?**	1 = Breastmilk only	
		2 = Breastfeeding as well as other milk (including formula) and/or food	
		3 = Milk or food only. No breastmilk	
B6	Today were you given any advice on how to feed your baby?<br>**Bakunikile namhlanje izeluleko mayelana nendlela yokudlisa noma ukuncelisa umntwana wakho?**	1 = Yes	2 = No<br>**→ Skip to B8**
B7	What advice were you given?<br>**Bakunikeze ziphi izeluleko?**<br><br>***Tick any mentioned by the mother***	1 = advised about breastfeeding technique	
		2= assisted with breastfeeding problems	
		3 = Advised to give only breastmilk to the baby	
		4 = How to express breastmilk if you are away from your baby	
		5 = Correct complementary (solid) foods to give the baby	
		6 = How many times per day to feed the baby	
		7= how much food to give the baby (portion size)	
		8 = to feed the child actively	
		9 = how to clean utensils bottles etc or other hygiene	
		10 = other	
B8	Today were you given any food supplements for your baby?<br>**Ngabe umntwana banunikezile yini izakha mzimba namuhla?**	1 = Yes	2 = No
B9	Today were you asked about how your baby is developing?<br>**Bakubuzile namhlanje ukuthi umntwana wakho ukhula kanjani?**<br>*Note: i.e. smiling, sitting, words responds to sounds (depends on child's age)*	1 = Yes	2 = No

C□□M□□□

B10	Today did your baby receive vitamin A drops (capsule squeezed into baby's mouth)<br>**Umntwana wakho uwatholile yini amaconsi e Vitamin A namuhla?**<br>*Note describe what Vitamin A drops look like*	1 = Yes	2 = No	3 = DNK
B11	Today did your baby receive an immunisation?<br>**Ugomile namuhla umntwana?**	1 = Yes	2 = No	
B12	Today were you asked about your baby's health?<br>**Ubuziwe mayelana nempilo yomntwana wakho namhlanje?**	1 = Yes	2 = No	
B13	Today was your baby referred to the hospital?<br>**Ngabe bamudluliselile yini umntwana e sibedlela namuhlanje?**	1 = Yes	2 = No<br>→ **Skip to B16**	
B14	What is the reason for the referral?<br>**Yini eyenze ukuthi umntwana bamudlulisele esibhedlela?**	1= malnutrition or low weight<br>2 = other		
B15	Give the reason for referral?<br>**Yini eyenze ukuthi umntwana bamudlulisele esibhedlela?**			
B16	Today were you asked about your own health?<br>**Bakubuzile ngempilo yakho namhlanje?**	1 = Yes	2 = No	
B17	Today were you asked if you were coughing?<br>**Bakubuzile ukuthi uyakhwehlela namhlanje?**	1 = Yes	2 = No	
B18	Today did anyone talk to you about having a PAP smear?<br>**Ukhona useke wakhulumisana nawe mayelana ne-PAP smear namuhla?**	1= Yes	2 = No	
B19	Today were you offered a PAP smear?<br>**Unikeziwe yini ithuba lokwenza i-PAP smear namuhla?**	1 = Yes	2 = No	
B20	Have you ever had a PAP smear?<br>**Usuke wayenza yini i-PAP smear ngaphambilini?**	1 = Yes<br>2 = No → **Skip to B22**<br>3 = Do not know → **Skip to B22**		
B21	When was your last PAP smear?<br>**Wagcina nini ukwenza i PAP smear?**<br>*Note ask the mother to provide her best estimate of when she last had a PAP smear*	1 = Today<br>2 = In the last 12 months<br>3 = More than 12 months ago		
B22	Today were you asked if you had a vaginal discharge or sores in your vaginal area?<br>**Bakubuzile ukuthi uphuma idischarge noma unezilonda esithweni sangasese?**	1 = Yes	2 = No	
B23	Today did anyone talk to you about family planning?<br>**Bakubuzile namhlanje ukuthi uyakusebenzisa ukuhlelwa komndeni?**	1 = Yes	2 = No	
B24	Today were you offered family planning?<br>**Bakubuzile namhlanje ukuthi uyakudinga ukuhlelwa komnbdeni?**	1 = Yes	2 = No	
B25	Are you currently using any family planning?<br>**Ngabe uyawuhlela yini umndeni?**	1 = Yes<br>→ **Skip to B27**	2 = No	
B26	Would you have accepted family planning if offered today?<br>**Ukube bewinikeziwe ithuba lokuhlela umndeni ubuzohlela namuhla?**	1 = Yes<br>→ **Skip to B30**	2 = No<br>→ **Skip to B30**	
B27	What family planning are you on?<br>**Usebenzisa yiphi indlela yokuhlela umndeni?**<br>*Select the option for condoms only if this is the only form of family planning used. If it is being combined with any other method select the other method.*	1= Injection<br>2= Oral contraceptive<br>3= IUCD/Loop → **Skip to B30**<br>4= Implant → **Skip to B30**<br>5 = condoms only → **Skip to B30**		

C□□ M□□□

B28	When did you last receive your family planning?<br>**Ugcine nini ukuzohlela/ukuhlela umndeni umndeni?**<br><br>*If the mother has her appointment card, ask to check the dates*	1 = Today → **Skip to B30**<br>2 = Last 14 days<br>3 = More than 2 weeks ago<br>4 = Do not know	
B29	When is your next family planning visit?<br>**Uzophinde uhlele nini umndeni futhi?**	1 = In the next 14 days<br>2 = More than 2 weeks from today<br>3 = Do not know	
B30	Today were you asked if you needed condoms?<br>**Bakubuzile namuhla ukuthi uyawandinga yini ama condom?**	1 = Yes	2 = No
B31	Today did you take condoms?<br>**Uwatholile/thathile ama-condom namuhla?**	1 = Yes → **Skip to C1**	2 = No
B32	Would you have accepted condoms if you had been offered?<br>**Ukuba bewunikeziwe ama condom, ubuzowathatha yini?**	1 = Yes	2 = No

**SECTION C: POSTNATAL CARE**			
check	Is this a 6weeks visit?	1 = Yes	2 = No → **Skip to D1**
C1	Did you have a postnatal check for yourself and your baby?<br>**Baku-checkile yini wena nomntwana wakho emva kokuthi ubelethile?**	1 = Yes	2 = No
C2	Did you have your blood pressure checked today?<br>**Bakucheckile yini I-BP?**	1 = Yes	2 = No
C3	Were your breasts examined?<br>**Bawaholile yini amabele akho namuhla?**	1 = Yes	2 = No
C4	Did you have a sample of blood taken today (other than for an HIV test)?<br>**Ukhona owezempilo othahte igazi kuwena emunweni?**	1 = Yes	2 = No
C5	Did the health worker test a urine sample for the mother?<br>**Bawuhlolile yini umchamo wakho?**	1 = Yes	2 = No
C6	Did you have an examination of the vaginal area today?<br>**Ukewahlolwa isitho sangasese namuhla?**	1 = Yes	2 = No
C7	Did the health worker feel your abdomen?<br>**Ukuthintile yini, wezwa isisu sakho owezempilo?**	1 = Yes	2 = No
C8	Did the health worker observe you breastfeeding your baby?<br>**Ngabe owezempilo ukubhekile yini wabona ukuthi umntwana undlisa kanjani?**	1 = Yes	2 = No
C9	Did the health worker look inside the baby's mouth?<br>**Ngabe owezempilo ubhekile yini umlonyeni womntwana?**	1 = Yes	2 = No
C10	Did the health worker check the baby's umbilicus?<br>**Uyibhekile yini inkaba yomntwana owezempilo?**	1 = Yes	2 = No

C☐☐M☐☐☐

**SECTION D: HIV and PMTCT**			
Interviewer: now I am going to ask some questions about HIV			
D1	Today did anyone talk to you about HIV?<br>**Ngabe ukhona okhulume nawe nge sandulela ngculazi namuhla?**	1 = Yes	2 = No
D2	Today did the health worker ask you about your own HIV status?<br>**Ukhona okubuzile mayelana nesimo sakho segciwane lesandulela ngculaza?**	1 = Yes	2 = No
D3	Have you ever been tested for HIV (during your pregnancy or another time)?<br>**Usuke wahlolelwa igciwane lesandulela ngculaza (ngezikhathi ukhulelwe noma ngesinye isikhathi)**	1 = Yes	2 = No<br>→ **Skip to D18**
D4	When was your last HIV test<br>**Ugcine nini ukuhlolela i HIV?**	1= today<br>→ **Skip to D20**	2= previous
D5	When was your last HIV test?<br>*Note do not include if HIV test done today. If test done today or the mother refuses to answer write 999:9999*	☐☐☐/☐☐☐☐<br>M M M Y Y Y Y	
D6	What was the result of your last HIV test?<br>**Yayithini imiphumela yakho yokuhlolelwa igciwane lesandulela ngculaza kamuva?**	1 = Positive<br>2 = Negative → **Skip to C18**	3 = refuses to answer
D7	Were you on the PMTCT programme during your last pregnancy?<br>**Wawukhona kuloluhlelo olubheka ukuvimbela umama angatheleli umntwana wakhe ngegciwane lesandulela ngculaza (PMTCT) ekukhulelweni kwakho kamuva?**	1 = Yes	2 = No
D8	Are you currently on ART/FDC?<br>**Uyayisebenzisa imishanguzo?**	1 = Yes	2 = No<br>→ **Skip to D11**
D9	Did you receive your ART/ FDC medication today?<br>**Ngabe uyitholile yini imushanguzo namuhla?**	1 = Yes<br>→ **Skip to D11**	2 = No
D10	When is your next appointment to collect FDC/ART?<br>**Uzoza nini ukuzolanda ama ART/FDC futhi?**	1= in the next 7days<br>2 = more than 7days	
D11	Has your baby had a PCR test *before* today?<br>**Ngabe umntwana bake bamenza i-PCR?**<br>*Note blood taken from heel and dripped onto paper*	1 = Yes<br>→ **Skip to D14**	2= No
D12	Today was your baby offered a PCR test?<br>**Bamunikile umntwana ithuba lokwenziwa iPCR namhlanje?**	1 = Yes	2= No
D13	Did your baby have a PCR test today?<br>**Ngabe umntwana bamenzile i PCR namuhla?**<br>*Note blood taken from heel and dripped onto paper*	1 = Yes<br>→ **skip to D20**	2 = No<br>→ **skip to D20**
D14	Have you got the result of the PCR test?<br>**Uyitholile imiphumela ye PCR?**	1 = Yes	2 = No<br>→ **skip to D20**
D15	What is the result of the PCR test?<br>**Ithini imiphumela ye PCR?**	1= Positive<br>2 = Negative → **skip to D20**<br>3 = Do not know → **skip to D20**	
D16	Is your baby on ARVs?<br>**Umntwana wakho uyayithola imishanguzo?**	1 = Yes	2 = No<br>→ **skip to D20**

C☐☐ M☐☐☐

D17	Did your baby receive ARVs today?	1 = Yes<br>→ **skip to D20**	2 = No<br>→ **skip to D20**
D18	Today were you offered an HIV test?	1 = Yes	2 = No
D19	Today would you have accepted an HIV test if you had been offered?	1 = Yes	2 = No
D20	How many health workers did you see during your visit today?<br><br>*Note include all health workers who provided any care to the mother or baby during this visit*	☐☐	
D21	During your visit today were you able have a discussion with the health worker about private matters?<br>**Ibikhona namhlanje indawo obungakhuluma nomsebenzi wasekliniki ngasese?**<br>*Note did the mother feel that she had an opportunity to raise any private matter with the health worker?*	1 = Yes<br>→ **Skip to E1**	2 = No
D22	Why were you not able to speak to the health worker privately?<br><br>***More than one response allowed***	1 = More than one mother/child carer present	
		2 = More than one health worker present	
		3 = Other	

C□□M□□□

Section E: ROAD TO HEALTH CARD (RTHC)				
**Interviewer: Do you have your child's RTHC? May I please see it**				
E1	Does the mother have a RTHC for the child	1 = Yes		2 = No<br>→Skip to F22
E2	Has the birth weight been filled in (page 6)	1 = Yes		2 = No
E3	Is birth length filled in? (page 6)	1 = Yes		2 = No
E4	Is head circumference at birth filled in? (page 6)	1 = Yes		2 = No
E5	Has the weight been recorded for today?	1 = Yes		2 = No
E6	Has the length been recorded for today?	1 = Yes		2 = No
E7	Has the weight been plotted for today on the weight for age graph (pg 14 &15)	1 = Yes		2 = No<br>**→Skip to E9**
E8	How is the weight for age?	1= on or above -2 line<br>2= between -2 line and -3 line<br>3= below -3 line		
E9	Has the weight for height been plotted for today? (p18)	1 = Yes		2 = No<br>**→Skip to E11**
E10	How is the weight for height?	1= on or above -2 line<br>2= between -2 line and -3 line<br>3= below -3 line		
E11	Has a diagnosis been made in regards to growth or nutrition?<br>*Look on the clinical notes section starting on page 21 as well as on the well child visits recording sheet on Page 2. Look for 'growing well' 'not growing well' or 'severe malnutrition, as well as for 'SAM' or 'MAM'*	1 = Yes		2 = No<br>**→Skip to E13**
E12	What diagnosis been made in regards growth or nutrition<br>*Note check the consultation record*	1= growing well<br>2= not growing well<br>3 = Moderate acute malnutrition (MAM)<br>4 = Severe malnutrition or severe acute malnutrition (SAM)<br>5 = NAM at risk<br>6 = other malnutrition		
E13	Has the child been referred to the hospital today?<br>*Note check the consultation record*	1 = Yes		2 = No
E14	Comment:<br>*Note only complete if more explanation is required for your answers*			
DEVELOPMENT (p13)				
E15	Has the developmental screening been recorded at 14 weeks?	1 = Yes	2 = No	3 = NA
E16	Has the developmental screening been recorded at 6 months?	1 = Yes	2 = No	3 = NA
E17	Has the developmental screening been recorded at 9 months?	1 = Yes	2 = No	3 = NA

C□□M□□□

E18	Has the developmental screening been recorded at 18 months?	1 = Yes	2 = No	3 = NA
	IMMUNISATIONS (p5)			
E19	Have immunisations been given at 6weeks?	1 = Yes	2 = No	3 = NA
E20	Have immunisations been given at 10weeks?	1 = Yes	2 = No	3 = NA
E21	Have immunisations been given at 14weeks?	1 = Yes	2 = No	3 = NA
E22	Have immunisations been given at 9months?	1 = Yes	2 = No	3 = NA
E23	Have immunisations been given at 18months?	1 = Yes	2 = No	3 = NA
HIV (p 8)				
E24	Is the mother's latest HIV test results filled in? (page 7)	1 = Yes		2 = No
E25				
E26	If positive, has the child had a PCR taken? (page 8)	1 = Yes	2 = No	3 = NA (HIV neg)
E27	If yes, has the PCR result been filled in? (page 8)	1 = Yes	2 = No	3 = NA (HIV neg)
VITAMIN A (p9)				
E28	Has the Vitamin A supplementation at 6months been recorded? (page 9)	1 = Yes	2 = No	3 = NA
E29	Has the Vitamin A supplementation at 12months been recorded?	1 = Yes	2 = No	3 = NA
E30	Has the Vitamin A supplementation at 18 months been recorded?	1 = Yes	2 = No	3 = NA
DEWORMING (p9)				
E31	Has the deworming treatment been recorded for 12 months?	1 = Yes	2 = No	3 = NA
E32	Has the deworming treatment bee recorded for 18 months?	1 = Yes	2 = No	3 = NA

C□□ M□□□

Section F: WEIGHT AND HEIGHT MEASUREMENTS			
WEIGHT (as recorded on RTHC)<br>*Note when no more weights recorded record 9's in all boxes*			
F1	Record birth weight		□□□□ g
F2	Weight 1 recorded	□□/□□□/□□□□	□□□□ g
F3	Weight 2 recorded	□□/□□□/□□□□	□□□□ g
F4	Weight 3 recorded	□□/□□□/□□□□	□□□□ g
F5	Weight 4 recorded	□□/□□□/□□□□	□□□□ g
F6	Weight 5 recorded	□□/□□□/□□□□	□□□□ g
F7	Weight 6 recorded	□□/□□□/□□□□	□□□□ g
F8	Weight recorded today		□□□□ g
HEIGHT (as recorded on RTHC)			
F9	Length at birth		□□□ cm
F10	Length 1	□□/□□□/□□□□	□□□ cm
F11	Length 2	□□/□□□/□□□□	□□□ cm
F12	Length 3	□□/□□□/□□□□	□□□ cm
F13	Length 4	□□/□□□/□□□□	□□□ cm
F14	Length 5	□□/□□□/□□□□	□□□ cm
F15	Length 6	□□/□□□/□□□□	□□□ cm

C□□M□□□

MUAC measurements			
F16	MUAC 1	□□/□□□/□□□□	□□.□ cm
F17	MUAC 2	□□/□□□/□□□□	□□.□ cm
F18	MUAC 3	□□/□□□/□□□□	□□.□ cm
F19	MUAC 4	□□/□□□/□□□□	□□.□ cm
F20	MUAC 5	□□/□□□/□□□□	□□.□ cm
F21	MUAC 6	□□/□□□/□□□□	□□.□ cm
Height and weight measurement today			
F22	Weight measured today		□□□□ g
F23	Length measured today		□□□ cm
F24	MUAC measured today		
